# Effectiveness of school-based physical activity programs in enhancing attention, academic performance, and social relationships among children with intellectual disabilities: evidence from Pakistani schools

**DOI:** 10.3389/fpsyg.2024.1431890

**Published:** 2024-09-10

**Authors:** Saima Sabri, Mei-Yue Zhang, Lu Guo, Junhua Dang, Zhi-Xiong Mao

**Affiliations:** ^1^School of Psychology, Beijing Sport University, Beijing, China; ^2^Institute of Social Psychology, School of Humanities and Social Sciences, Xi’an Jiaotong University, Xi’an, China; ^3^Department of Surgical Sciences, Uppsala University, Uppsala, Sweden

**Keywords:** intellectual disability, physical activity, behavioral problems, attention, academic performance

## Abstract

Physical activity (PA) offers extensive benefits for all children, including those with intellectual disabilities (ID), who face significant challenges in behavioral management and psycho-social well-being. This study investigates the effects of school-based PA on attention, academic performance, and relationships with teachers and parents in children with ID. A 12-week single-blind randomized controlled trial was conducted with 102 children with ID, aged 6 to 12 years (71 boys and 31 girls) from grades 1 to 5. Participants were divided into three groups: MVPA (moderate to vigorous PA), MPA (mild PA), and NPA (no PA). Each group engaged in PA three times a week for 45 min per session, with activities planned by a fitness trainer and supervised by the researcher. Outcome measures were assessed using SNAP-IV, STRS, CPRS, and APRS scales before and after the intervention. The results indicated that MVPA had a more significant positive impact on all outcomes compared to MPA and NPA. MPA also produced notable improvements relative to NPA. These findings underscore the importance of integrating PA into educational settings as a comprehensive strategy to enhance attention, academic performance, and social interactions for children with ID. This research highlights PA as a vital tool for addressing behavioral challenges and fostering better developmental outcomes in this population.

## Background

Intellectual disability (ID) is one of the most common types of disability (American Psychiatric Association, 2013) and is defined by below-average mental ability and a lack of the analytical, social, and functional abilities required for normal living [American Association on Intellectual and Developmental Disability (AAIDD), 2010]. Great endeavor has been put into designing various intervention programs promoting healthier development of children and adolescents with ID, with a special focus on enhancing parent-sensitive responsiveness and improving cognitive and social outcomes of those with ID in school settings (Guralnick, 2011; Guralnick, 2017). Given the benefits of consistent physical activity (PA) on one’s physical and mental health have been firmly established (Kristen et al., 2017; Sabri et al., 2023), researchers have begun to investigate the interventive efficiency of PA for children and adolescents with ID (Jin et al., 2018; Salehpoor et al., 2015). A recently meta-analysis including 15 studies revealed that PA could dramatically improve psychological health (e.g., decreasing anxiety and depression while increasing self-esteem, with an effect size of Hedges’ *g* = 0.54) and cognitive functions (e.g., attention and inhibitory control, with an effect size of Hedges’ *g* = 1.24) of children and adolescents with ID, suggesting PA is a valuable interventive approach treating ID (Yang et al., 2022).

School-based physical activity (PA) programs, such as physical education (PE), offer a promising approach to supporting children with disabilities (Baron and Faubert, 2005; Filazoğlu-Çokluk et al., 2015; Nicholson et al., 2011; Savucu and Biçer, 2009). Research indicates that sports and motor activities in school can enhance cognitive abilities in children with intellectual disabilities (ID), including memory, attention, and executive skills (Goldshtrom et al., 2010; Javan et al., 2014; Protic and Válková, 2018). Racket-sport interventions, in particular, have been shown to improve visual perception and executive functions in children with ID (Chen et al., 2015). However, whether these cognitive benefits translate into academic success remains an open question, as there are few studies directly examining the impact of PA on academic achievement. Existing evidence suggests that PA positively influences academic success in ID students, as they tend to be more engaged in classwork following PA sessions (Dandashi et al., 2015; Everhart et al., 2012). Beyond cognitive benefits, sports participation also addresses social skill deficits common among children with ID. These children often struggle with social skills, which can limit their involvement in social situations (Eriksson et al., 2007). Engaging in PA can improve social interactions and relationships for children with ID (Siperstein et al., 2009). Specifically, participation in school sports can enhance social competence and foster growth in these children (Brooks et al., 2015), and PA can lead to positive changes in their home and social environments (Savaş et al., 2016). Conversely, a lack of PA or an inactive lifestyle can diminish social cohesion and competence (Mehmet et al., 2018).

Despite evidence suggesting the positive impact of PA on mental and behavioral improvements in children with ID, existing studies exhibit several significant methodological limitations. For instance, there is a scarcity of randomized controlled trials, which hampers the ability to draw causal inferences. Additionally, many studies focus on only one type of ID, limiting the generalizability of their findings. Exercise programs in these studies often lack details regarding the types of exercises, their durations, and repetitions, making it challenging to replicate or compare outcomes. Furthermore, most research has been conducted in developed countries or territories, such as France and Hong Kong, raising questions about the applicability of these findings to developing or underdeveloped countries like Pakistan. These methodological shortcomings highlight a significant research gap and underscore the need for more comprehensive and rigorously designed studies to better understand how PA influences the healthy development of children with ID.

Therefore, the current paper aims to fill this gap by employing a randomized controlled trial design to test 1) whether the beneficial effect PA on cognitive functions such as attention could be generalized to an ID sample from Pakistan; 2) whether such effect could transfer to academic performance; and 3) whether PA also brings positive effect for children and adolescents with ID on social interaction skills manifested as improved relationship with their teachers and parents.

## Methods

### Participants

The present study was conducted in three special schools in Rahim Yar Khan, Punjab Pakistan (Rahim Yar Khan, Sadiqabad, and Khanpur District). A total of 102 participants from grades 1 to 5, with an age range of 6–12, enrolled in the study. All individuals diagnosed with intellectual disabilities (ID) by qualified school personnel, according to DSM-5 criteria, exhibit characteristics such as an IQ score of 70 or lower, significant impairments in intellectual functioning (including reasoning, problem-solving, planning, abstract thinking, and both academic and experiential learning), and deficits in adaptive functioning that result in failure to meet socio-cultural standards (e.g., lack of judgment) associated with intellectual impairments. Initially, 353 participants were considered for the study. However, 251 were excluded for the following reasons: (1) 81 did not meet DSM-5 criteria for ID; (2) 23 had previously participated in PA or exercise programs; (3) 51 were unwilling to participate; (4) 39 were outside the required age range; (5) 48 had family-related concerns that could affect participation; and (6) 9 were using medication that could potentially impair physical performance. Ultimately, 102 participants were selected for the experiment, comprising 61 individuals with a diagnosis of Mild ID and 41 individuals with a diagnosis of Moderate ID. This sample was carefully chosen to ensure accurate representation across the specified ages and educational levels. Detailed characteristics of the study sample are provided in the Supplementary Table S1.

**Figure 1:**
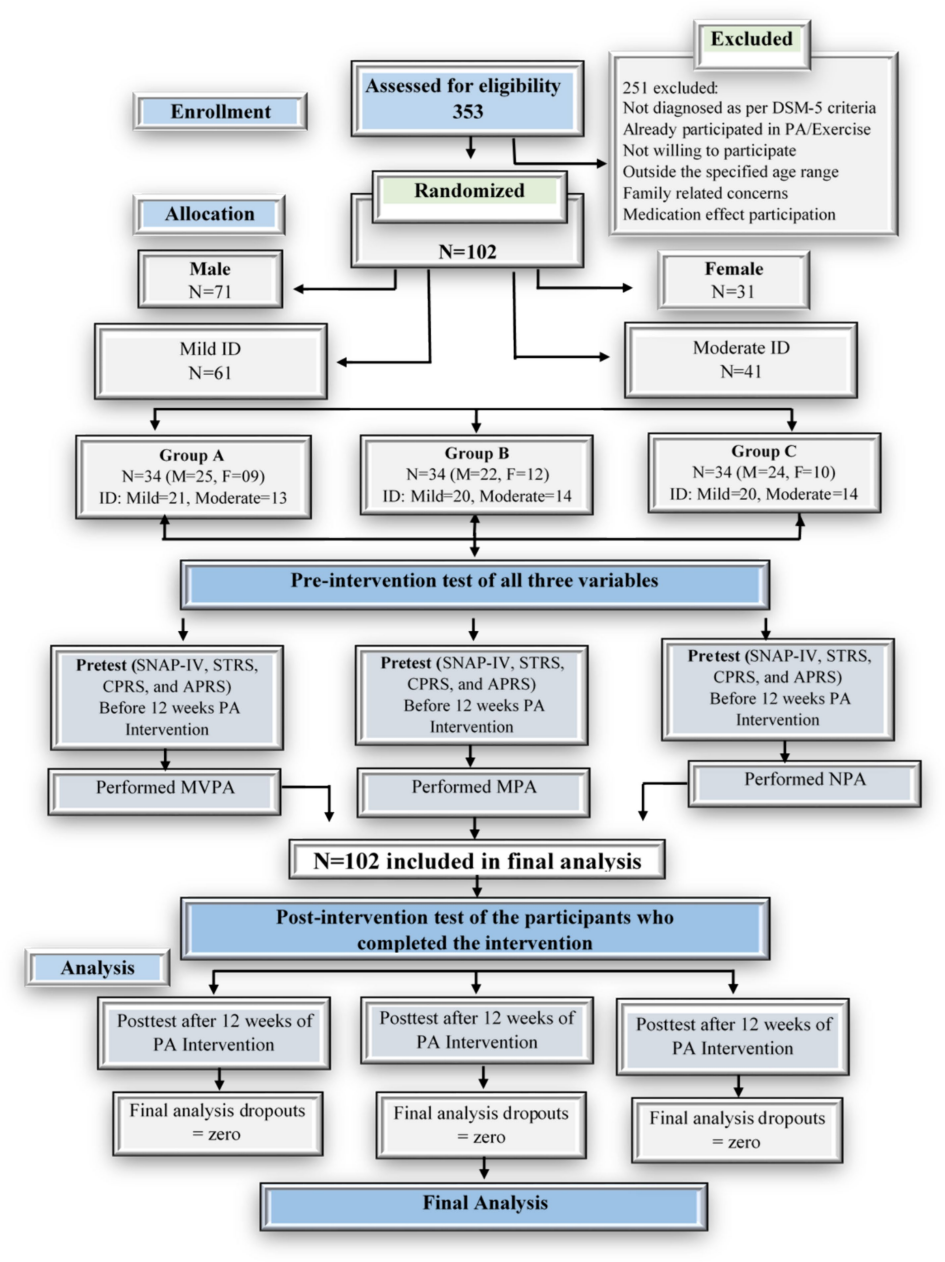
CONSORT flow diagram of research participants. ID, Intellectual Disability; PA, Physical Activity; SNAP-IV, Swanson, Nolan, and Pelham, Version IV scale; STRS, Student-Teacher Relationship Scale; CPRS, Child–Parent Relationship Scale; APRS, Academic Performance Rating Scale; MVPA, Moderate to Vigorous Physical Activity; MPA, Mild Physical Activity; NPA, No Physical Activity.

Permission was obtained from the relevant authorities, including the local District Education Directorate, school management, and teachers. The study was approved by the ethics committee of Beijing Sport University. Additionally, informed consent was secured from the parents of all participating children. Figure 1 illustrates the flow chart of the study.

### Study design and procedure

**Figure 2:**
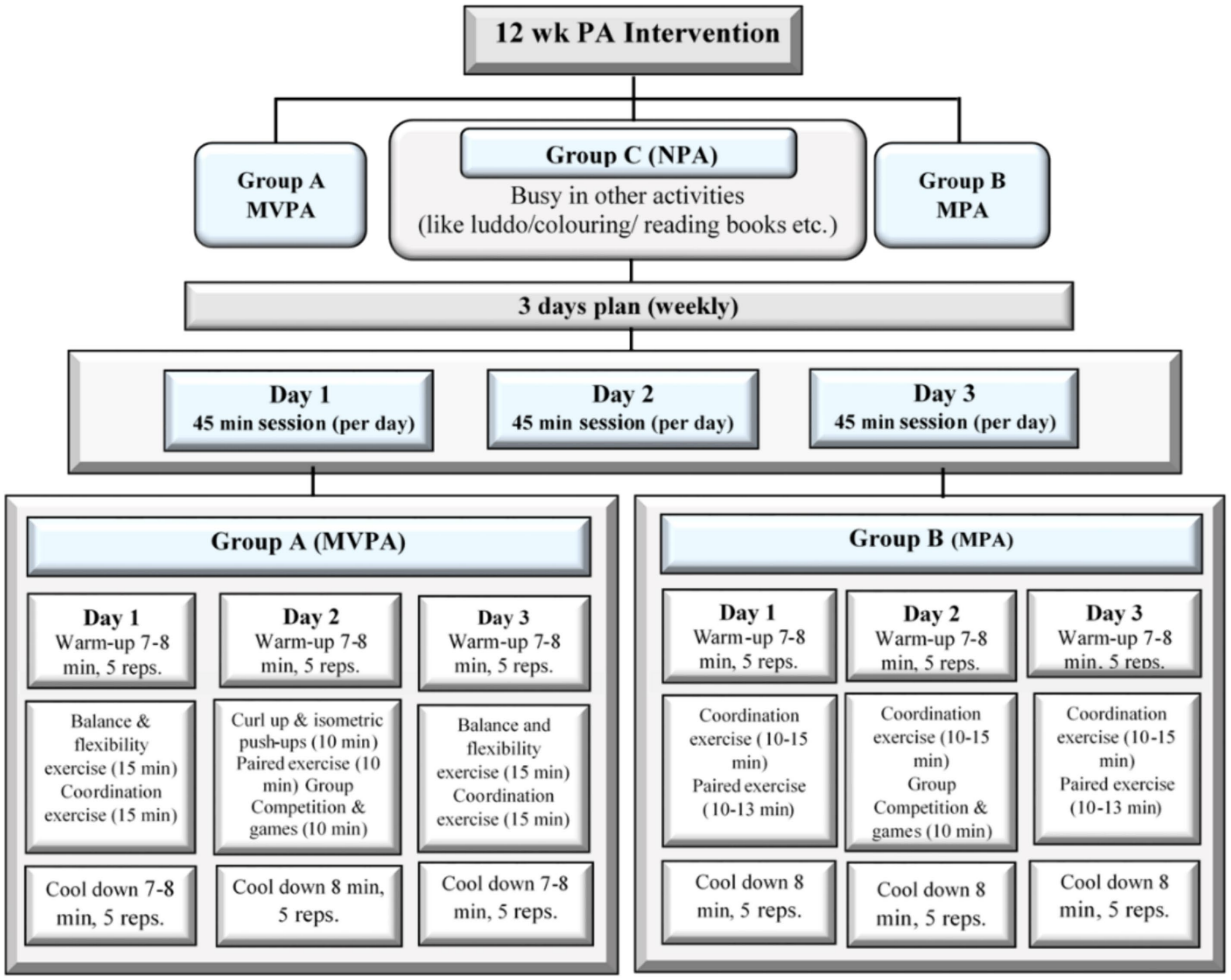
Brief description of the PA intervention plan for each group of participants.

With a single-blind 12-week randomized controlled trial design, this study examined the effect of a school-based PA intervention program on behavioral improvements in attention, academic performance, and student-teacher relationship in children with ID. Participants were divided into three groups and matched by age, grade, and level of ID (i.e., mild or moderate). One group received a moderate to vigorous physical activity (MVPA) intervention; one group received a mild physical activity (MPA) intervention; and the final group, as the control group, did not receive a physical activity intervention (NPA). The exercise program was designed and executed by a fitness trainer and monitored by the researcher. In particular, the 12-week PA intervention was executed 3 days per week, 45 min per day. Classification of exercise groups (MVPA & MPA) was determined on the basis of the selection, intensity, repetitions, duration and number of exercises performed each specified day. Exercise intensity was determined using the talk test, a simple method for measuring relative intensity. It is a valid, accurate, realistic, and cost-effective method for administering and tracking exercise intensity (Reed and Pipe, 2014). The intervention plan for the three groups is shown in Figure 2, which includes warm-up and cool-down exercises, balancing and flexibility exercises, modified curl-up and isometric pushups, coordination exercises, paired exercises, and group competitions and games. Details of the exercises performed by the MVPA group and the MPA group according to exercise type and intensity are given in the Supplementary material. Valid instruments were applied to parents and teachers before and after the PA program to measure dependent variables.

### Instruments

*Swanson, Nolan, and Pelham, Version IV scale* (SNAP-IV) was completed by both teachers and parents to determine the level of attention of ID children. The SNAP-IV is widely used to screen for attention deficit and hyperactivity disorder (ADHD) (Swanson, 1992; Swanson et al., 1983). It consists of 26 items, and each item is scored on a four-point scale: 0 = not at all, 1 = just a little, 2 = quite a bit, and 3 = very much. The questionnaire is designed for children aged 6 to 18 years and takes approximately 10 min to complete (Costa et al., 2018; Yang et al., 2014). Although the SNAP-IV is frequently used in school-based, non-clinical settings (Bussing et al., 2008), current data also supports its validity in children with ID. Research has demonstrated strong psychometric properties, including high reliability and concurrent validity, as evidenced by positive correlations with other ADHD rating scales (Michael et al., 2004a,b). Additionally, the SNAP-IV subscales exhibit adequate internal consistency, which supports their use in this population and underscores their potential for broad applicability (Zieff et al., 2023).

Academic performance was measured by the *Academic Performance Rating Scale* (APRS), a teacher report scale used to measure the academic performance of a child with ID (Topor et al., 2010). The APRS consists of 19 items on which that teachers rate a child’s academic abilities and behaviors in the classroom on a scale ranging from 1 (never or poor) to 5 (quite common or excellent) (e.g., “What is the quality of this child’s reading skills?”).

The *Student-Teacher Relationship Scale* (STRS) was used to measure the relationship between ID children and their teachers (Sanja, 2017; Seven and Ogleman, 2014). The STRS consists of 15 self-report items completed by teachers that use a 5-point Likert scale to gain an understanding of a teacher’s relationship with a student, the interpersonal conduct of a student with the teacher, and the confidence of a teacher in knowing the feelings of the student toward the teacher (e.g., “This child and I always seem to be struggling with each other.”). Low scores indicate less conflicts and better relationships.

The *Child–Parent Relationship Scale* (CPRS) was used to measure the relationship between ID children and their parents (Driscoll and Pianta, 2011; Dyer et al., 2017). The CPRS is a self-report instrument consisting of 15 items completed by parents who evaluate their relationship with their child on a 5-point Likert scale (e.g., “My child is uncomfortable with physical affection or touch from me.”). Low scores indicate less conflicts and better relationships.

### Statistical analysis

A series of 2 (Intervention group: MVPA vs. MPA vs. NPA; Between-subject) by 2 (Time: Pretest vs. Posttest; Within-subject) ANOVAs with scores on SNAP-IV, APRS, STRS, and CPRS as dependent variables were conducted by using the SPSS. If an interaction were found, the simple effects test and post-hoc comparisons were then conducted to examine the specific pattern.

## Results

Table 1 shows the descriptive statistics of pretest and posttest scores on SNAP-IV, APRS, STRS, and CPRS total points and subscales. For SNAP-IV, a lower score indicates better attention, and a higher score indicates more attention deficiency. A decrease in scores from pretest to posttest means improvement of attention. For APRS, a higher score indicates better academic performance. For STRS and CPRS, a lower score indicates less conflict and a better relationship. A decrease in scores from pretest to posttest indicates an improvement in the relationship. A series of 2 (Intervention group: MVPA vs. MPA vs. NPA; Between-subject) by 2 (Time: Pretest vs. Posttest; Within-subject) ANOVAs with repeated measures were conducted by using the SPSS.

**Table 1:** Descriptive statistics of SNAP-IV, APRS, STRS, and CPRS.

SNAP-IV	SNAP-IV (TR)		SNAP-IV (PR)	
	Pretest	Post test	Pretest	Post test
MVPA (*n* = 34)	41.82 ± 2.66	33.79 ± 2.90	41.88 ± 2.99	33.88 ± 3.19
MPA (*n* = 34)	41.85 ± 2.60	37.29 ± 3.47	42.03 ± 2.70	37.35 ± 3.69
NPA (*n* = 34)	42.00 ± 2.13	41.65 ± 2.41	42.03 ± 2.33	41.82 ± 2.15

APRS	APRS	
	Pretest	Post test
MVPA (*n* = 34)	46.06 ± 4.59	50.41 ± 4.60
MPA (*n* = 34)	46.06 ± 4.80	48.09 ± 5.36
NPA (*n* = 34)	46.00 ± 2.09	46.38 ± 2.48

	STRS (TR) & CPRS (PR)	
	Pretest	Post test
STRS		
MVPA (*n* = 34)	48.82 ± 2.66	42.76 ± 3.52
MPA (*n* = 34)	48.20 ± 3.05	44.44 ± 3.72
NPA (*n* = 34)	45.79 ± 1.90	45.58 ± 2.07
CPRS		
MVPA (*n* = 34)	49.23 ± 2.74	42.91 ± 3.66
MPA (*n* = 34)	47.76 ± 2.42	44.35 ± 2.41
NPA (*n* = 34)	45.79 ± 2.05	45.50 ± 2.19

TR, Teachers’ ratings; PR, Parents’ ratings.

### Changes in teacher-reported attention deficiency (SNAP-IV)

The ANOVA with teacher-reported scores on SNAP-IV yielded a significant main effect of time, *F* (1, 99) = 241.18, *p* < 0.001, *η_p_^2^* = 0.71, a main effect of intervention group, *F* (2, 99) = 25.19, *p* < 0.001, *η_p_^2^* = 0.34, and an interaction between time and intervention group, *F* (2, 99) = 63.84, *p* < 0.001, *η_p_^2^* = 0.56. The simple effects test found that attention deficiency decreased significantly from pretest to posttest in the MVPA group, *F* (1, 99) = 278.53, *p* < 0.001, *η_p_^2^* = 0.74, and the MPA group, *F* (1, 99) = 89.79, *p* < 0.001, *η_p_^2^* = 0.48. However, attention deficiency remained unchanged in the NPA group, *F* (1, 99) = 0.54, *p* = 0.465, *η_p_^2^* = 0.01. Post-hoc comparison showed that the decrease of the MVPA group was higher than the other two groups (*p*s < 0.001) and the decrease of the MPA group was also higher than the NPA group (*p* < 0.001), indicating both PA groups gained attention improvement and the MVPA group gained the highest.

### Changes in parent-reported attention deficiency (SNAP-IV)

The ANOVA with parent-reported scores on SNAP-IV yielded very similar results, a significant main effect of time, *F* (1, 99) = 228.47, *p* < 0.001, *η_p_^2^* = 0.70, a main effect of intervention group, *F* (2, 99) = 22.17, *p* < 0.001, *η_p_^2^* = 0.31, and an interaction between time and intervention group, *F* (2, 99) = 63.18, *p* < 0.001, *η_p_^2^* = 0.56. The simple effects test found that attention deficiency decreased significantly from pretest to posttest in the MVPA group, *F* (1, 99) = 264.32, *p* < 0.001, *η_p_^2^* = 0.73, and the MPA group, *F* (1, 99) = 90.32, *p* < 0.001, *η_p_^2^* = 0.48. However, attention deficiency remained unchanged in the NPA group, *F* (1, 99) = 0.18, *p* = 0.677, *η_p_^2^* = 0.00. Post-hoc comparison showed that the decrease of the MVPA group was higher than the other two groups (*p*s < 0.001) and the decrease of the MPA group was also higher than the NPA group (*p* < 0.001).

### Changes in academic performance (APRS)

The ANOVA with teacher-reported scores on APRS revealed a significant main effect of time, *F* (1, 99) = 53.05, *p* < 0.001, *η_p_^2^* = 0.35. The main effect of intervention group was not significant, *F* (2, 99) = 2.37, *p* = 0.099, *η_p_^2^* = 0.05. However, the interaction between time and intervention group was significant, *F* (2, 99) = 12.94, *p* < 0.001, *η_p_^2^* = 0.21. The simple effects test found that academic performance increased significantly from pretest to posttest in the MVPA group, *F* (1, 99) = 64.22, *p* < 0.001, *η_p_^2^* = 0.39, and the MPA group, *F* (1, 99) = 13.96, *p* < 0.001, *η_p_^2^* = 0.12. However, academic performance remained unchanged in the NPA group, *F* (1, 99) = 0.75, *p* = 0.388, *η_p_^2^* = 0.01. Post-hoc comparison showed that the increase of the MVPA group was higher more than the MPA group (*p* = 0.003) and the NPA group (*p* < 0.001). The increase of the MPA group was also higher than the NPA group (*p* = 0.045).

### Changes in student-teacher relationship (STRS)

The ANOVA with teacher-reported scores on STRS yielded a significant main effect of time, *F* (1, 99) = 146.66, *p* < 0.001, *η_p_^2^* = 0.60, and an interaction between time and intervention group, *F* (2, 99) = 38.04, *p* < 0.001, *η_p_^2^* = 0.44. The main effect of intervention group was not significant, *F* (2, 99) = 0.60, *p* = 0.549, *η_p_^2^* = 0.01. The simple effects test found that STRS scores decreased significantly from pretest to posttest in the MVPA group, *F* (1, 99) = 160.56, *p* < 0.001, *η_p_^2^* = 0.62, and the MPA group, *F* (1, 99) = 61.99, *p* < 0.001, *η_p_^2^* = 0.39. However, STRS scores remained unchanged in the NPA group, *F* (1, 99) = 0.19, *p* = 0.668, *η_p_^2^* = 0.00. Post-hoc comparison showed that the decrease of the MVPA group was higher than the other two groups (*p*s < 0.001) and the decrease of the MPA group was also higher than the NPA group (*p* < 0.001), indicating both PA groups gained relationship improvement and the MVPA group gained the highest.

### Changes in child–parent relationship (CPRS)

The ANOVA with parent-reported scores on CPRS yielded a significant main effect of time, *F* (1, 99) = 137.85, *p* < 0.001, *η_p_^2^* = 0.58, and an interaction between time and intervention group, *F* (2, 99) = 37.38, *p* < 0.001, *η_p_^2^* = 0.43. The main effect of intervention group was not significant, *F* (2, 99) = 0.41, *p* = 0.665, *η_p_^2^* = 0.01. The simple effects test found that CPRS scores decreased significantly from pretest to posttest in the MVPA group, *F* (1, 99) = 164.40, *p* < 0.001, *η_p_^2^* = 0.62, and the MPA group, *F* (1, 99) = 47.86, *p* < 0.001, *η_p_^2^* = 0.33. However, CPRS scores remained unchanged in the NPA group, *F* (1, 99) = 0.36, *p* = 0.552, *η_p_^2^* = 0.00. Post-hoc comparison showed that the decrease of the MVPA group was higher than the other two groups (*p*s < 0.001) and the decrease of the MPA group was also higher than the NPA group (*p* < 0.001), indicating both PA groups gained relationship improvement and the MVPA group gained the highest.

## Discussion

The current paper employed a randomized controlled trial design to test whether the PA has a positive effect on cognitive functions in children with ID living in Pakistan, whether such effect could transfer to academic performance, and whether PA also improves their social interaction skills. The results revealed significant differences between pre-and posttest scores on the SNAP-IV, APRS, STRS, and CPRS after PA execution, indicating a significant improvement in the level of attention, academic performance, student-teacher relationship, and child–parent relationship.

Previous studies have consistently showed the beneficial effect of PA on cognitive functions (e.g., attention and inhibitory control) in developed countries/territories, with a large average effect (*g* > 1.00 or *η_p_^2^* > 0.20) (Yang et al., 2022). In our study conducted in a Pakistani sample, the significant changes in scores on SNAP-IV in both MVPA and MPA groups compared to the NPA group showed that engaging ID children in PA produced a great positive effect on their level of attention, which replicated previous findings in the domain of cognitive functions with a comparable level of effect size. Meanwhile, a more pronounced score difference in the MVPA group suggested that the level of attention improvement was closely related to the intensity of PA. Based on these findings, it is highly encouraging to integrate PA into the lives of individuals with ID to improve their physical and cognitive well-being.

Furthermore, we found the beneficial effect of PA could also transfer from attention to academic performance. However, the mechanism of such behavioral modification is not clear and requires further investigation to fully understand the link between PA and academic performance. According to some researchers, PA lowers levels of stress and anxiety, which in turn improves attention to complete academic tasks, complete schoolwork, and learn more effectively (Ibis and Aktug, 2018; Yılmaz and Soyer, 2018). Alternatively, others also pointed out the crucial role of PA interventions in promoting the development of intelligence and self-control of school-aged children (Tomporowski et al., 2007; Farhangi and Alamdarloo, 2015).

Finally, according to the teachers and the parents of the ID children who participated in the study, there was a substantial drop between pre and posttest scores on the STRS and CPRS, with a lower score indicating a better relationship and less conflict. Higher STRS and CPRS score disparities between the MVPA and NPA groups compared to the differences between the MVPA and MPA groups also demonstrated that PA intensity had a significant impact on the degree of improvement in ID children’s relationships with their teachers and parents. Although MVPA had the greatest favorable effect, the significance of MPA cannot be overlooked, therefore both therapies are useful and can improve the degree of social relationships. Taken together, these results showed that school games have a favorable impact on children with ID’s quality of life as well as their physical, social, cognitive, and emotional development, which increases our confidence that participation in daily PA can be utilized as an alternate method of coping with the behavioral and emotional problems of children with ID.

Our research stands out in the field due to several distinct strengths compared to similar studies. In terms of internal validity, we implemented a rigorous participant selection process, including only children who had not participated in any PA programs in the months leading up to the study. This approach effectively controls for confounding factors related to prior PA levels (Farhangi and Alamdarloo, 2015). Our study achieved a 0% dropout rate, demonstrating exceptional participant retention and engagement, which contrasts with high dropout rates observed in previous research (Savaş et al., 2016) and ensures the consistency of our data. Additionally, unlike prior studies (Everhart et al., 2012), our PA program was led by a single fitness trainer for all participants, minimizing variability and ensuring uniformity in training. Regarding external validity, we designed a school-based PA intervention to evaluate its impact on children with ID. By integrating PA into classroom activities, our approach aims to engage ID students during school hours, which has significant practical implications (Protic and Válková, 2018; Everhart et al., 2012). We meticulously detailed each exercise program, including both mild and vigorous PA, to provide a comprehensive understanding of which types of exercise yield the most significant effects. This level of detail is often overlooked in similar research (Ilkım et al., 2018). These combined features enhance the reliability and validity of our study, making a substantial contribution to the field of PA interventions for children with ID.

Future research should expand the geographical scope to include all districts of Pakistan, which will help address regional differences and enhance the generalizability of the findings. Additionally, studies should involve participants from a wider age range beyond just primary school students to assess whether the effects of PA remain consistent across different developmental stages. Comparative studies are also recommended to examine the impact of PA on children with various disabilities to determine if the benefits are similar across different special needs groups.

Moreover, future studies should explore a variety of exercise modalities to identify which types of exercise produce the most significant behavioral improvements in children with ID. Longitudinal research is necessary to evaluate the long-term effects of PA on behavioral changes. Additionally, tailored PA programs should be designed and tested to determine the most effective strategies for enhancing behavioral outcomes within classroom and school settings. Addressing these areas will provide a more comprehensive understanding of how PA influences behavioral and academic achievement, ultimately leading to more effective interventions and educational methods.

Our findings also offer valuable insights for a range of professionals, including sports psychologists, physiotherapists, researchers, sports coaches, clinical psychologists, and psychiatrists. For sports psychologists and coaches, the research provides strategies to enhance student engagement and performance. Physiotherapists can adapt their therapies to support behavior modification, while clinical psychologists and psychiatrists can incorporate PA into their treatment plans. Researchers will benefit from a solid foundation for future research, promoting a multidisciplinary approach to improving outcomes for students with special needs.

## Conclusion

Our results indicate that PA can significantly enhance attention, academic performance, and social relationships among children with ID. The PA intervention yielded promising outcomes, with notable improvements observed across all measured variables. This suggests that school-based PA programs are effective in boosting the well-being of children and adolescents with ID.

However, further experimental research is needed to identify the optimal combination of exercise types and durations. It is advisable for educational institutions to incorporate PA into their curricula and provide targeted training for educators to facilitate successful implementation. Additionally, fostering multidisciplinary collaboration among parents, teachers, and healthcare professionals will enable the development of personalized PA interventions. This approach can enhance educational outcomes and promote a more inclusive learning environment for students with ID.

## Data availability statement

The datasets presented in this study can be found in online repositories. The names of the repository/repositories and accession number(s) can be found at: https://osf.io/es8ax/?view_only=1518767499c2492985d91a88b7f0d0d0.

## Ethics statement

The studies involving humans were approved by School of Psychology, Beijing Sport University. The studies were conducted in accordance with the local legislation and institutional requirements. Written informed consent for participation in this study was provided by the participants’ legal guardians/next of kin.

## Author contributions

SS: Conceptualization, Data curation, Formal analysis, Investigation, Methodology, Writing – original draft. M-YZ: Validation, Writing – review & editing. LG: Supervision, Validation, Writing – review & editing. JD: Formal analysis, Supervision, Validation, Writing – review & editing. Z-XM: Conceptualization, Project administration, Supervision, Validation, Writing – review & editing.
