# Supplementary material for: Effectiveness of school-based physical activity programs in enhancing attention, academic performance, and social relationships among children with intellectual disabilities: evidence from Pakistani schools
Source: Front Psychol. 2024 Sep 10;15:1431890. doi: 10.3389/fpsyg.2024.1431890 (PMC11420793; doi:10.3389/fpsyg.2024.1431890)
Supplement: Supplementary file 1 [file Data_Sheet_1.docx]

**Supplemental Materials for**

**Beneficial Impact of School Based Physical Activity on Attention, Academic Performance, and Social Relationships and for Children with Intellectual Disability**

**1. Group A (MVPA)**

Group A (N=34) having mild to moderate level of ID, were performed MVPA for 3 days in a week and 45 minutes a day. We made two exercise plans which performed on alternate days after warm-up and cool-down exercise. Day one plan were started by doing warm-up exercises for 7 to 8 minutes with 5 repetitions. After warm-up exercises fitness trainer guided and teaches participants to perform balance and flexibility exercises. The approximated time duration for balance and flexibility exercises was 15 minutes. After balance and flexibility exercises, participants engaged to do coordination exercises. The expected time duration for coordination was also 15 minutes. After coordination exercises the participants did cool down exercise for 7 to 8 minutes with 5 repetitions. Day 2 plan was also started by doing warm-up exercises directed by fitness trainer for 7 to 8 minutes a day with 5 repetitions. After warm-up exercises children’s with ID guided and demonstrated by fitness trainer to perform modified curl up and isometric pushups for 10 minutes. After modified curl up and isometric pushups session the children’s were engaged in paired exercises for the duration of 10 minutes. After paired exercises participants also engaged in group competitions and games. The estimated time duration for group competitions was 10 minutes a day. Cool down exercises were performed for approximately 7 to 8 minutes with 5 repetitions.

**1.1 Warm up exercises**

The warm-up exercises are the physical and mental preparations to engage in an exercise and activity. Warm-up exercises help the body to prepare itself for exercise and to reduce the injury risks. Some of the warm up exercises that were designed by fitness trainer and performed by children’s with ID are listed below

- Arms overhead
- Arms side & up
- Arm circles
- Hand claps
- Extended arm & fingers
- Arms forward & up
- Hands overhead
- Standing body twist
- Spread wings
- Jumping

**1.2 Balance and Flexibility exercises**

Balance and flexibility exercises are among the family including strength and endurance necessary for healthy workout routine. Balance exercises helps to improve the ability to control and stabilize body position while flexibility is the ability of range of a joint or group of joints to move joints effectively over a complete range of motion. Balance and flexibility exercises that designed by fitness trainer and performed by children’s with ID are listed below

- Triangle pose right
- Triangle pose left
- Forward bend
- Quad stretch right
- Quad stretch left
- Trunk side flexion
- Trunk left side bending
- Trunk rotation

**1.3 Paired exercises**

Pairing exercises is a popular program design strategy. Paired exercise is act of executing two exercises of opposing muscle groups back to back during a workout. Usually paired exercises are designed in order to increase the effectiveness of a training session.

In the current study the paired exercises that designed by fitness trainer and performed by children’s with ID are listed below

- Seesaw
- Double boat
- Lean on me
- Back to back twist
- Moved around circle

**1.4 Modified curl up and isometric pushups**

Curl up is one of the most common abdominal exercises. This is done to strengthen the abdominal muscles and to firm up the abdominal region. Push‐ups are very common exercise that can be assimilated into a training program in order to gain the upper body strengthen.

In the current study the modified curl up and isometric pushup exercises that designed by fitness trainer and performed by children’s with ID are listed below

- Rag doll
- Down dog
- Plank
- Camel
- Child pose
- Cobra pose
- Bridging
- Pushups
- River pose
- Butterfly stretch

**1.5 Co-ordination exercises**

Coordination play vital role in sports and in day-to-day living. A coordination exercise is the ability to perform smooth, precise and well-ordered motor responses and it involves the ability to select the right muscle at the right time with appropriate intensity to accomplish proper action. Coordination exercises that are performed by ID children’s and designed by fitness trainer are listed below

- Hell to knee slide
- Catching balls
- Scissor step
- Stair climbing
- Hurdle walk

**1.6 Group competition & games**

Group competitions & games are those in which players play against one another. Group competitions & games are the structured form of play usually performed for enjoyment and occasionally used as an educational tool. Group competitions and games that designed by fitness trainer and performed by children’s with ID are listed below

- Tug of war
- Running
- Throwing & catching
- Football
- Cricket

**1.7 Cool down exercises**

These include exercises that help the body transition from working hard to relaxing. These exercises allow heart and breathing rate to return to normal and relaxation. Cool down exercises that designed by fitness trainer and performed by children’s with ID are listed below

- Jogging
- Knee to chest stretch right
- Knee to chest stretch left
- Lunge stretch right
- Lunge stretch left
- Chill pose

**2. Group B (MPA)**

Group B (N=34) having mild to moderate level of ID, were performed MPA for 3 days in a week and 45 minutes a day. Like group A, we also made two exercise plans which performed on alternate days after warm-up with cool-down exercise. Day one plan were started by doing warm-up exercises for 7 to 8 minutes with 5 repetitions. After warm-up exercises fitness trainer guided and teaches participants to perform coordination exercises. The estimated time duration for coordination exercises was 10 to 15 minutes. After coordination exercises, the participants were guided to do paired exercises in order to increase the productivity and rewards of the exercise session. The expected time duration that was spend in paired exercises for each day was 10 to 15 minutes. After paired exercises the participants were directed and guided by to performed cool down exercises in order to normalize the heart and breathing rates. The time duration for cool down exercises was 7 to 8 minutes with 5 repetitions. Day 2 plan was also started by doing warm-up exercises directed by fitness trainer for 7 to 8 minutes a day with 5 repetitions. After warm-up exercises children’s with ID guided and demonstrated by fitness trainer to perform coordination exercises. The estimated time duration for coordination exercises was 10 to 15 minutes. After coordination exercises, the participants were guided to play group competitions and games. The expected time spend in group competitions and games for one day was 10 to 15 minutes. Cool down exercises were performed for approximately 7 to 8 minutes with 5 repetitions to relax the body and heart rate.

**2.1 Warm up exercises**

The warm-up exercises that designed by fitness trainer and performed by children’s with ID are listed below

- Arms overhead
- Arms side & up
- Arm circles
- Hand claps
- Extended arm & fingers
- Arms forward & up
- Hands overhead
- Standing body twist
- Spread wings

**2.2 Coordination exercises**

In the current study the coordination exercises that designed by fitness trainer and performed by children’s with ID are listed below

- Hell to knee slide
- Catching balls
- Stair climbing

**2.3 Paired exercises**

In the current study the paired exercises that designed by fitness trainer and performed by children’s with ID are listed below

- Seesaw
- Back to back twist
- Moved around circle

**2.4 Group competitions & games**

In the current study the group competitions and games that designed by fitness trainer and performed by children’s with ID are listed below

- Running
- Throwing & catching
- Football

**2.5 Cool down exercises**

Cool down exercises that designed by fitness trainer and performed by children’s with ID are listed below

- Jogging
- Knee to chest stretch right
- Knee to chest stretch left
- Lunge stretch right
- Lunge stretch left
- Chill pose

**Results**

Descriptive statistics have been carried out to observe the distribution of the data. Table S1 presents the list of the concise statistics.

**Table S1 Demographic data of study participants**

| **Variables** | **Group A (N=34)** | **Group B (N=34)** | **Group C (N=34)** |
| --- | --- | --- | --- |
| Age (*M*, SD) | 9.76(1.89) | 9.58(1.84) | 9.73(1.88) |
| Gender (*n*, %) |  |  |  |
| Boys | 25(73.5) | 22(64.7) | 24(70.6) |
| Girls | 9(26.5) | 12(35.3) | 10(29.4) |
| ID Diagnosis (*n*, %) |  |  |  |
| Mild | 21(61.8) | 20(58.8) | 20(58.8) |
| Moderate | 13(38.2) | 14(41.2) | 14(41.2) |

The study sample consisted of 102 participants. Age (*M*, SD) for group A, B & C participants was 9.76 (1.89), 9.58 (1.84) and 9.73 (1.88) respectively. Relative percentage of boys / girls was 25 (73.5%) / 9 (26.5%), 22 (64.7%) / 12 (35.3%) and 24 (70.6%) / 10(29.4%) for group A, B & C respectively. Moreover, percentage of mild / moderate ID participants was 21 (61.8%) / 13 (38.2%) for group A, 20 (58.8%) / 14 (41.2%) for group B and 20 (58.8%) / 14 (41.2%) for group C participant.
